# Comparing Distribution of Harbour Porpoises (*Phocoena phocoena*) Derived from Satellite Telemetry and Passive Acoustic Monitoring

**DOI:** 10.1371/journal.pone.0158788

**Published:** 2016-07-27

**Authors:** Lonnie Mikkelsen, Frank F. Rigét, Line A. Kyhn, Signe Sveegaard, Rune Dietz, Jakob Tougaard, Julia A. K. Carlström, Ida Carlén, Jens C. Koblitz, Jonas Teilmann

**Affiliations:** 1 Department of Bioscience, Aarhus University, Roskilde, Denmark; 2 AquaBiota Water Research, Stockholm, Sweden; 3 Swedish Museum of Natural History, Stockholm, Sweden; 4 Coalition Clean Baltic, Uppsala, Sweden; 5 German Oceanographic Museum, Stralsund, Germany; 6 BioAcoustics Network, Stralsund, Germany; CNRS, FRANCE

## Abstract

Cetacean monitoring is essential in determining the status of a population. Different monitoring methods should reflect the real trends in abundance and patterns in distribution, and results should therefore ideally be independent of the selected method. Here, we compare two independent methods of describing harbour porpoise (*Phocoena phocoena*) relative distribution pattern in the western Baltic Sea. Satellite locations from 13 tagged harbour porpoises were used to build a Maximum Entropy (MaxEnt) model of suitable habitats. The data set was subsampled to one location every second day, which were sufficient to make reliable models over the summer (Jun-Aug) and autumn (Sep-Nov) seasons. The modelled results were compared to harbour porpoise acoustic activity obtained from 36 static acoustic monitoring stations (C-PODs) covering the same area. The C-POD data was expressed as the percentage of porpoise positive days/hours (the number of days/hours per day with porpoise detections) by season. The MaxEnt model and C-POD data showed a significant linear relationship with a strong decline in porpoise occurrence from west to east. This study shows that two very different methods provide comparable information on relative distribution patterns of harbour porpoises even in a low density area.

## Introduction

Visual observations from plane or boat have long been the primary method to monitor cetaceans at sea [1]. As this still holds true for some species (e.g. [2–4]), methods such as satellite telemetry (e.g. [5–6]) and acoustic monitoring (e.g. [7–9]) are increasingly being used to describe distribution patterns and relative occurrences. Satellite telemetry reveals the movement patterns of the tagged animals with spatial and temporal resolution depending on programming of the tags and use of satellite systems, e.g. GPS system within tens of meters and Argos system within hundreds of meters [10]. With a high number of tagged individuals, the recorded movements may be extrapolated to estimate spatial patterns at population level (e.g. [6]). The draw backs from using satellite tracking are generally small sample sizes, auto-correlation, as one position is dependent on the previous one, presence only data, and spatial bias towards the tagging site, especially during short tracks. However, satellite positions can be used to model suitable habitats using the species distribution model MaxEnt (Maximum Entropy) [11–13]. Habitat suitability models can be useful when prioritizing between areas to reach certain conservation and management goals, especially in depleted stocks where distribution and abundance information is limited (e.g. [14–15]). Such models estimate the relationship between species records at specific locations and the environmental characteristics of the same sites [16]. MaxEnt was specifically developed for “presence only data” [13], which is necessary as “absence data” are unobtainable from satellite telemetry. The model works well with a relatively small sample size [17–18], as in the case of cetacean tagging research. Additionally, MaxEnt is robust towards positioning errors [19] and with inconsistency between spatial resolution of observations and environmental variables [20]. However, presence-only models also hold some limitations; they only provide estimates of relative suitability compared to occupancy probabilities obtained from presence-absence models [21]. Also, inherent data issues such as sample selection bias (bias in presence records) may severely affect model results [22].

Acoustic monitoring makes use of the echolocation clicks produced by toothed whales such as the harbour porpoise (*Phocoena phocoena*, L. 1758). Porpoises use high frequency narrow band echolocation almost continuously to navigate, socialize, and search for prey [23–25], making them ideal for automated detection with little risk of misclassification as their sounds have unique characteristics [26–27]. Static acoustic monitoring devices are advantageous in that they can be deployed for longer periods and provide continuous information on the presence of echolocating animals [28–30]. Still, these devices have relatively short maximum detection ranges of a few hundred meters, requiring several units to cover larger areas [9]. Acoustic data have mostly been used for relative estimates of density; however, recent studies have converted static acoustic data into density estimates [9, 31–32].

The method chosen for monitoring distribution patterns of a species is usually related to the specific question that needs to be answered, but different methods should reflect the same patterns in distribution, seasonal movements and abundance of animals to be valid. Here, we investigate whether two independent approaches, a MaxEnt habitat suitability prediction based on satellite tracks and the relative occurrence from acoustic data loggers, C-PODs (collected under the EU Life project, SAMBAH), from the same area, provide comparable results. It is the first time such data sets are compared directly, and also the first time detailed information on porpoise habitat use in the Western Baltic Sea is provided.

## Materials and Methods

### Satellite telemetry

Aarhus University (former National Environmental Research Institute, NERI) has since 1997 tagged harbour porpoises with Argos satellite transmitters in Danish waters. Porpoises incidentally caught in pound nets are tagged within 36 hours of entrapment. The tagging procedure was developed in cooperation with marine mammal veterinarians and is described in detail by Sveegaard et al. [6]. The tagging of harbour porpoises were approved by the Danish Animal Welfare Division, Ministry of Justice, permit number: 1995-101-62 and and 2010/561-1801, and carried out under the permission from the Danish Forest and Nature Agency, Ministry of Environment (SN 343/SN-0008).

**Fig 1:**
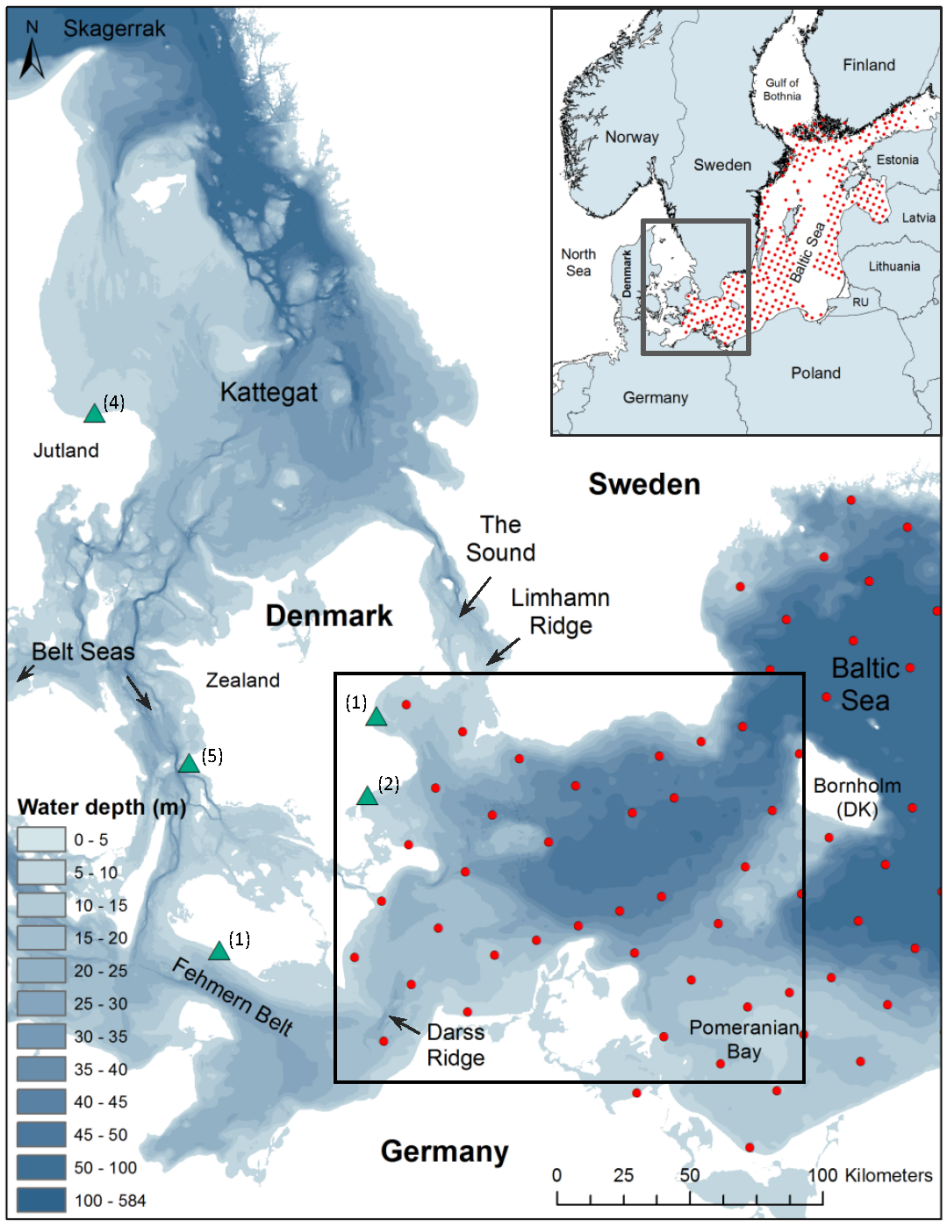
The study area in the south-western Baltic Sea. The study area was based on the spatial overlap between the satellite positions and the acoustic recording stations. Tagging locations of the 13 harbour porpoises that utilized the study area are indicated by green triangles. Numbers in brackets indicate the number of animals tagged in each location. The SAMBAH C-POD stations are shown as red dots, only the 36 stations inside the study area were included in the analysis.

Thirteen tagged porpoises utilised the study area in the south-western Baltic Sea during 2006 to 2012. Of these, three were tagged inside the study area and the rest moved into the study area after tagging (Fig 1). The south-western Baltic Sea constitutes the eastern range of the porpoises tagged in the inner Danish waters, hence the density of tagged animals is relatively low here [6]. It also constitutes the eastern range of the ‘Belt Sea’ population, separating it from the ‘Baltic Proper’ population [33].

The locations received from Argos were filtered by a SAS Argos-Filter v7.03 (D. Douglas, USGS, Alaska Science Center, Alaska, USA [34]). This filter is a Distance-Angle-Rate (DAR) filter and applies user-defined settings between successive locations for distance, turning angles and maximum swim speed to filter out the most unlikely locations. All filter settings were made according to Sveegaard et al. [6].

Satellite positions from the first 2 days after tagging were excluded to reduce spatial influence of the release site. The tags were programmed to only transmit every other day to save battery, but in order to further reduce autocorrelation within data, only one position (best location class and most uplinks) per animal per transmission day was selected for the analysis. By comparing the uncertainty provided by Argos (optional service) of the filtered and the unfiltered positions, showed that the precision of the average error radius was reduced from approx. 1400 m to 300 m. Positions were divided into four seasons (Dec-Feb, Mar-May, Jun-Aug, Sep-Nov). As most tags transmitted for more than three months they contributed to more than one season. To visualise positions for each season we used kernel density estimation (KDE) in the Geospatial Modelling Environment software v. 0.7.2.1 (SpatialEcology.com). The Gaussian kernel function was applied, bandwidth = SCV (Smooth Cross Validation) and cell size = 0.0005. The kernel estimates where then visualised using 10% intervals in contour lines in ArcGIS 10.2.1.

#### MaxEnt modelling

MaxEnt is available as a stand-alone Java program [13] through the statistical program R [35] using the package dismo (species distribution modelling) by Hijmans et al. [36]. The basic principle of the MaxEnt model is to compare environmental variables at the positions where porpoises have been observed (presence) with the environmental variables at a random selection of positions in the landscape (backgrounds) (for a detailed statistical description of MaxEnt see Elith et al. [37]). The number of backgrounds locations from the landscape was set to 10,000, to be sure that the environmental conditions in the area were met. In short, two probability densities are created, one estimated from the presence data and one from the landscape. MaxEnt then minimizes the relative entropy between the two probability densities, where the estimation of the predictor variables are formed in the same way as transforming variables in e.g. regression that are used to describe trends. The predictor variables are environmental factors that are believed to be relevant for the habitat of harbour porpoise distribution. We allowed only linear (L), quadratic (Q) and hinge (H) relationships (called features in MaxEnt) of the predictor variables in order to reduce the risk of overfitting. Furthermore, MaxEnt provides protection against overfitting via regularization, which penalizes the inclusion of additional predictor variables that results in little or no “gain” to the model [38]. In order to select the combination of the features and degree of regularization, we ran models with regularization values ranging from 0.5 to 4.0 (increments of 0.5) and combinations of the three features (L, Q, LQ, H, LQH) using the library ENMeval in R [39]. The model with the lowest Akaike Information Criterion (AIC) was chosen for further analyses. We evaluated the model by dividing the dataset into a training dataset (80% of the presence data), from which the model was developed, and a test dataset (20% of the presence data), from which the model was evaluated. Furthermore, we evaluated the variability of the model results by bootstrapping the complete dataset with 100 replications.

The MaxEnt predictions were evaluated by the mean AUC (Area Under the receiver operating Characteristics curve) value of the 100 bootstrap models. As there are no true absence data, AUC scores represent the probability that a randomly chosen presence location was more likely to have the species present, than a randomly selected pseudo-absence location chosen from the entire study area [13]. An AUC of 0.5 indicates that model performance is equal to that of a random prediction. Models with AUC above 0.75 are considered potentially useful, 0.80–0.90 good and 0.90–1.0 excellent [40–41].

The importance of the predictor variables was evaluated by the jack-knife test that measures firstly the gain when the variable is the only variable in the model, and secondly the decrease in gain if the variable is omitted from the full model.

#### Environmental variables

The environmental variables chosen as input to the model (Table 1) were believed to influence the harbour porpoise response to the environment. The physical variables could either be affecting the animals directly, indirectly by affecting prey occurrence, or serve as proxies of one or several other factors affecting the animals directly or indirectly (Table 1). It has been suggested that porpoise distribution follows that of its prey [42–43]. However, when no information exists on prey distribution, factors assumed to be proxies for prey occurrence are often used instead (e.g. [8]), and may even be more useful than prey occurrence at some scales [44–45]. Parameters describing seafloor characteristics as well as hydrodynamic variables have been shown to influence porpoise occurrence [42–43, 46–48]. Therefore, we chose to include the hydro dynamic variables current gradient (front), salinity, temperature and current velocity (Table 1). The dynamic variables were based on hourly time series modelled for the period 2002–2012 by the company Bolding & Burchard [49]. The static variables included were depth, slope, curvature of the bottom (convex or concave), distance to shoreline, sediment type as well as the number of ships taken from a random period (Table 1). The modelling of static variables as well as integrating the dynamic variables to create a spatial resolution of 0.5x0.5 km was done by the company DHI (www.dhigroup.com).

**Table 1:** Description of the environmental variables included in the model. The modelling of the dynamic variables was conducted by Buchart and Bolding Aps [4,9] and the modelling of static variables was done by DHI (www.dhigroup.com). All variables had a spatial resolution of 0.5x0.5 km.

	Environmental factor	Description	Source
**Static variables**	Depth	Depth of seafloor (m)	DHI
	Slope	Slope of the seafloor (degrees)	DHI
	Curvature	Relief of the sea floor, whether it is concave (negative value), convex (positive value) or flat (zero) in relation to the surrounding cells	DHI
	Distance to shoreline	Euclidian distance to the shoreline (m)	DHI
	Sediment type	Surface sediments classified as either bedrock, hard bottom complex, sand, clay or mud	http://www.helcom.fi/GIS/BalanceData
	Ship traffic	Number of ships (using mandatory AIS i.e. >50m) taken from a random period (Aug-Sep 2010) converted into an index with grids of maximum density set to 1.	Danish Maritime Authority (http://www.dma.dk/)
**Dynamic variables**	Front	Front as a measure for local surface horizontal gradient of currents (m/s/m)	Bolding&Burchard Aps / DHI
	Salinity	Local surface salinity (psu)	Bolding&Burchard Aps
	Temperature	Local surface water temperature (°C)	Bolding&Burchard Aps
	E-W velocity	Local surface E-W current velocity component (m/s)	Bolding&Burchard Aps
	N-S velocity	Local surface N-S current velocity component (m/s)	Bolding&Burchard Aps

### Acoustic detections

Static acoustic monitoring devices, C-PODs (Cetacean and Porpoise Detectors, Chelonia Ltd), were deployed as part of a large-scale EU LIFE+ funded project called SAMBAH (”Static Acoustic Monitoring of the Baltic Sea Harbour Porpoise” 2010–2015, www.SAMBAH.org). The C-PODs were positioned in a random systematic grid (with few alterations due to permission issues and trawling) with 25 km between each station in waters 5-80m deep. Data were collected from April 2011 until June 2013. In the present study, we used data from the 36 stations in the south-western Baltic (Fig 1). The C-PODs were serviced at intervals of 6 months to change batteries and download data. Some C-PODs were lost due to trawling while others were malfunctioning, so the data coverage is variable between stations, ranging from 104 to 738 days (see successful data acquisition in S1 Table).

The C-POD is a self-contained data logger that records properties of tonal signals, including the time and duration of echolocation signals made by harbour porpoises (and other toothed whales). The C-POD.exe software (http://www.chelonia.co.uk) uses an algorithm that isolates porpoise click trains (series of clicks divided by pauses) from other sounds [50–51]. A specific classifier (Hel1) was developed under the SAMBAH project for areas where the density of harbour porpoises is extremely low, such as in large parts of the Baltic Sea. We applied the Hel1 classifier to the data in C-POD.exe v2.043 and exported as Porpoise Positive Days (PPD) meaning the number of days where at least one porpoise click train was detected, and as Porpoise Positive Hours (PPH), the number of hours per day where at least one porpoise click train was detected. Data were presented as the number of positive days/hours per day relative to all monitoring days for the specific stations (% PPD/PPH). The results from the C-PODs were divided into the same seasons as the satellite data.

### Comparing satellite telemetry with acoustic detections

The estimated habitat suitability from MaxEnt (0.5x0.5 km, output resolution of the model) was averaged within a circle with a radius of 1 km around each C-POD position. MaxEnt pixels, where the centre was included in the 1 km radius was used to calculate a mean value (app. 12 pixels) and was plotted against the % PPD/PPH for that position. Pearson’s product-moment correlation was applied to test the relationship between MaxEnt averaged predicted values and the % PPD/PPH recorded at the respective C-POD stations.

## Results

### Satellite telemetry

**Fig 2:**
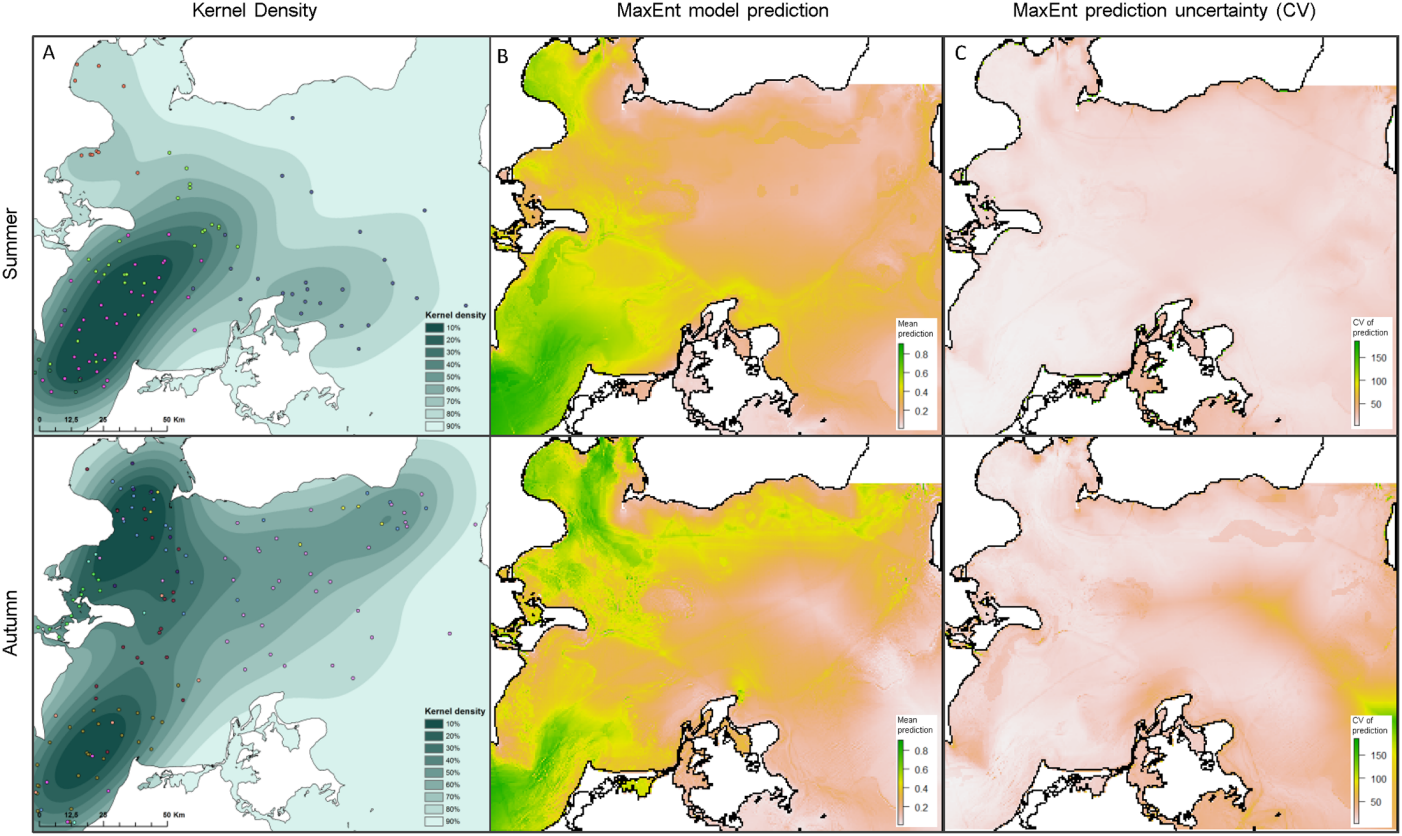
Kernel and MaxEnt results. (A) Kernel density results for summer (Jun-Aug, top row) and autumn (Sep-Nov, bottom row). (B) Mean prediction of the probability of presence of harbour porpoise based on 100 bootstrap models. The scale of the colouring can be interpreted as the relative probability of presence of harbour porpoise given the environment. (C) The uncertainty of the prediction expressed by the coefficient of variation (CV).

A total of 277 positions from 13 porpoises (from the period 2006–2012) were included in the model, distributed over the four seasons (Table 2). Transmitter life times ranged from 31 to 274 days (mean = 182). In spring and winter only 7 and 29 positions were available, which was not enough to construct a reliable model. Thus, only summer and autumn, with 102 and 139 positions, respectively, was used for further analysis. Summer results show a strong concentration of satellite positions in the south-western waters between Denmark and Germany (Fig 2A). In autumn, positions are clustered further north with high densities both in the south-west and in the north-west, in addition to a small concentration in the north-eastern waters. Both seasons displayed a strong gradient in density of positions from west to east (Fig 2A).

**Table 2:** Number of positions and individuals used in the MaxEnt model during the four seasons. Data from 13 individual harbour porpoises tagged during 2006–12 are included. Each tag only transmitted every other day to save battery. One individual may contribute to more than one season depending on the time of tagging and transmission lifetime of the tag.

Season	No. of positions used in model	No. of tagged individuals	Average no. of days in study area	Min/median/max number of positions per individual
Summer (Jun-Aug)	102	5	45	5/25/36
Autumn (Sep-Nov)	139	11	31	4/9/34
Winter (Dec-Feb)	29	5	10	1/3/6
Spring (Mar-May)	7	2	7	2/5/11

#### Model results

Preliminary runs of MaxEnt with different combinations of features and regularization parameters showed that the model with lowest AIC included linear and quadratic relationships with predictor variables in both seasons and with regularization parameters of 0.5 for the summer and 2.0 for the autumn season. The mean results of testing the 100 bootstrapped MaxEnt models, trained on 80% of the presence data and evaluated on the remaining 20%, were AUC values of 0.76 and 0.78 for summer and autumn, respectively.

During summer, the most suitable habitat for porpoises appears to be in the south-western part of the study area, in the waters between Denmark and Germany and along the Danish coast (Fig 2B). The uncertainty of the prediction expressed by the coefficient of variation (CV, Fig 2C) shows a small increase in uncertainty towards the north-eastern part of the study area. The most important predicted areas in autumn are in the western part, in the waters between Denmark and Germany as during summer, but also further north between Denmark and Sweden. The eastern and especially south-eastern part of the study area appears to be less suitable in autumn, coinciding with the highest uncertainty in the prediction. The seasonal shift in density of satellite positons to the north is also reflected in the model results (Fig 2A and 2B).

#### Environmental variables

**Fig 3:**
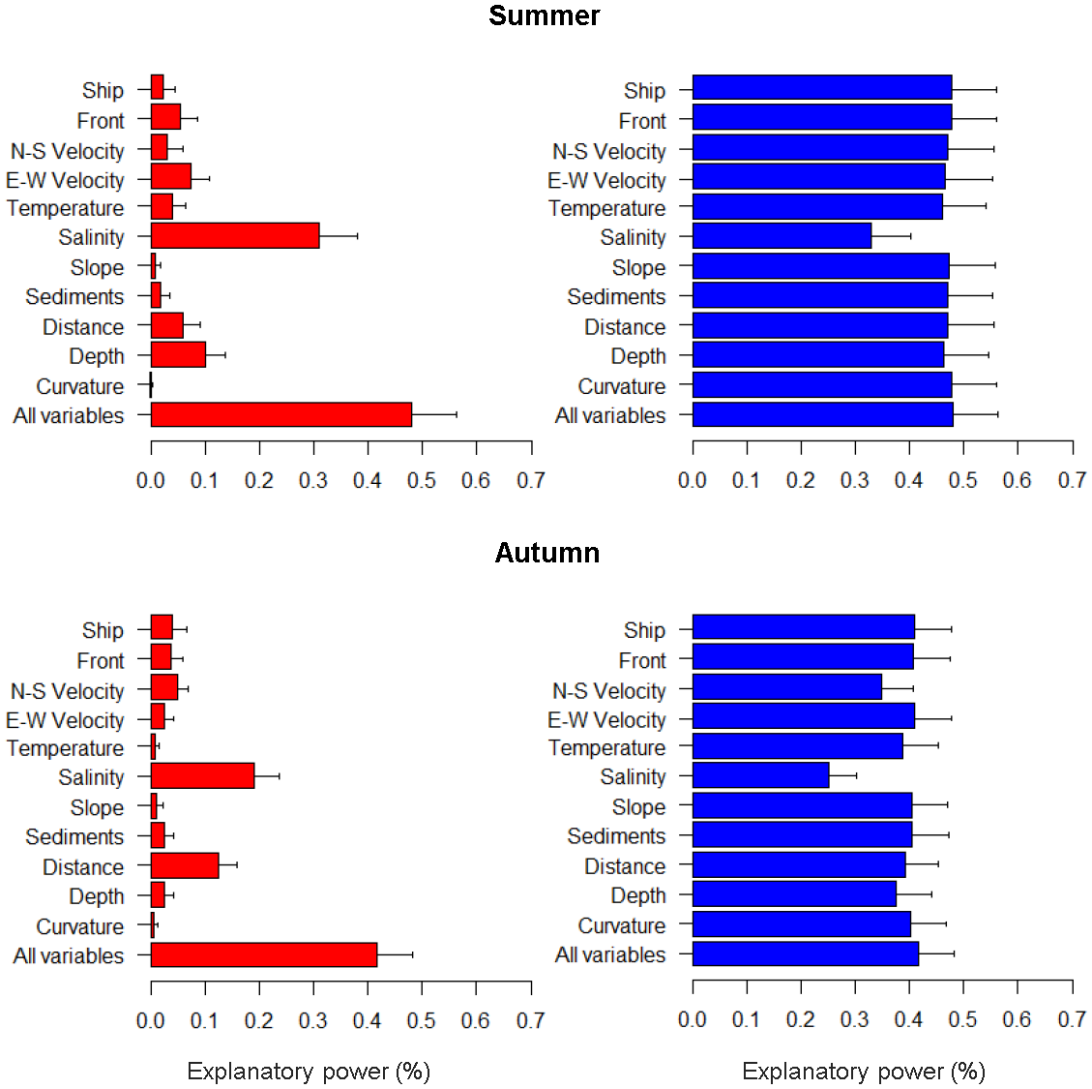
Jack-knife test. Results of the jack-knife test of variable importance after 100 bootstraps of the MaxEnt model. The bars indicate the explanatory power when only a single variable is included in the MaxEnt model (left/red) and the explanatory power when the variable is omitted from the full model (right/blue). Error bars represent standard deviation.

The results of the jack-knife showed that salinity in summer was the single most important variable when used in isolation, followed by depth, E-W current velocity and distance to shoreline (Fig 3). Salinity is also the variable that decreases the gain the most when omitted from the full model, emphasizing the importance of this variable. In autumn, salinity also clearly appears as the most important variable both when used as a single variable, and as gain decreased when omitted from the full model (Fig 3). Other variables with some degree of importance in autumn are distance to shoreline, when used as a single variable, N-S current velocity, and depth, when omitted from the full model.

**Fig 4:**
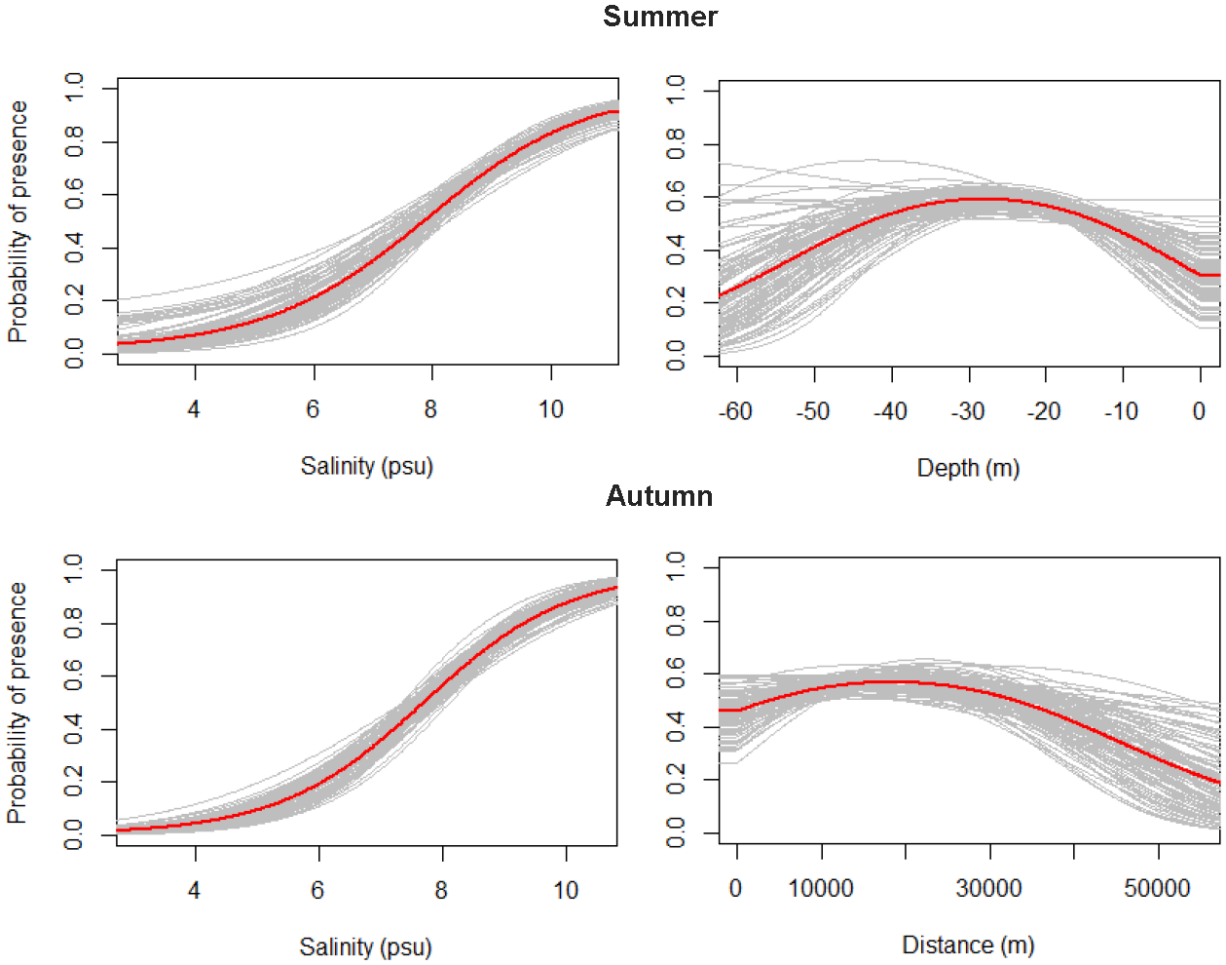
Response Curves. Response curves for the two most important explanatory variables given the model for summer and autumn. The curves show how the probability of presence changes as the value for a particular variable change, while keeping all other variables at their average sample value. Response curve for all 100 bootstrap models are shown with the mean curve in red.

The response curves for the two most important variables in summer and autumn given the model, illustrates the relationship between probability of presence and the environmental variables (Fig 4). In both seasons, the probability of presence increases with increasing salinity. The optimal depth in summer is around 20–30 meters which relates to a coastal occurrence in autumn between 10 and 30 km that drops with increasing distance.

### Acoustic detections

**Fig 5:**
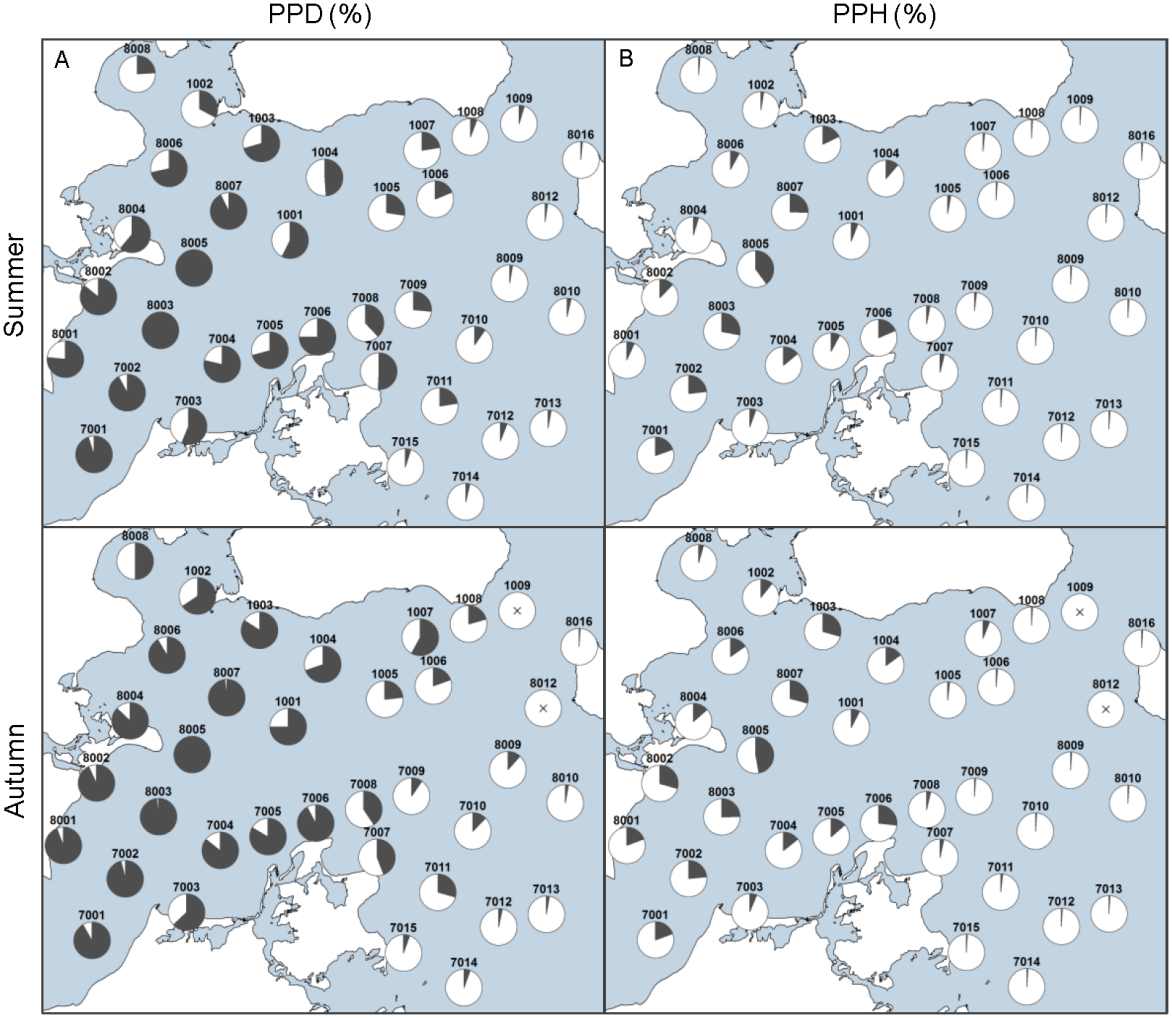
Results from the acoustic C-POD study. Stations are shown with station number and (A) the percentage of porpoise positive days by season (PPD %) and (B) the percentage of porpoise positive hours per days by season (PPH %). Numbers between 8001–8016 are Danish stations, 1001–1009 are Swedish stations, and 7001–7015 are German stations. Stations with X marks that no click were recorded at all in that period (station 8012 did not have any recordings for the autumn season due to loss of equipment, (S1 Table)).

The results from the C-POD study reveal a general decreasing gradient in detections from the south-western part of the study area towards the north and east (Fig 5). During summer, the majority of the stations located in the south-western area show 75–100% PPD, with a decreasing trend towards northern and eastern directions (Fig 5A). In autumn, there were in general more PPD on almost all stations in the study area, except for one station (1009) where no porpoises clicks were recorded at all in this period (station 8012 did not have any recordings from the autumn period due to loss of equipment). In both seasons, the most easterly stations display either zero or a very low proportion of detections. A similar pattern is seen in the average PPH, yet not as pronounced (Fig 5B). The stations, where the highest number of positive hours per day is found, are in the south-west decreasing towards the north and east. As with the PPD, there are in general more PPH recorded in autumn than in summer. The largest difference between the two data sets is that the PPH percentages, in general, are much lower and no station reaches 100%.

### Relationship between model and C-POD results

**Fig 6:**
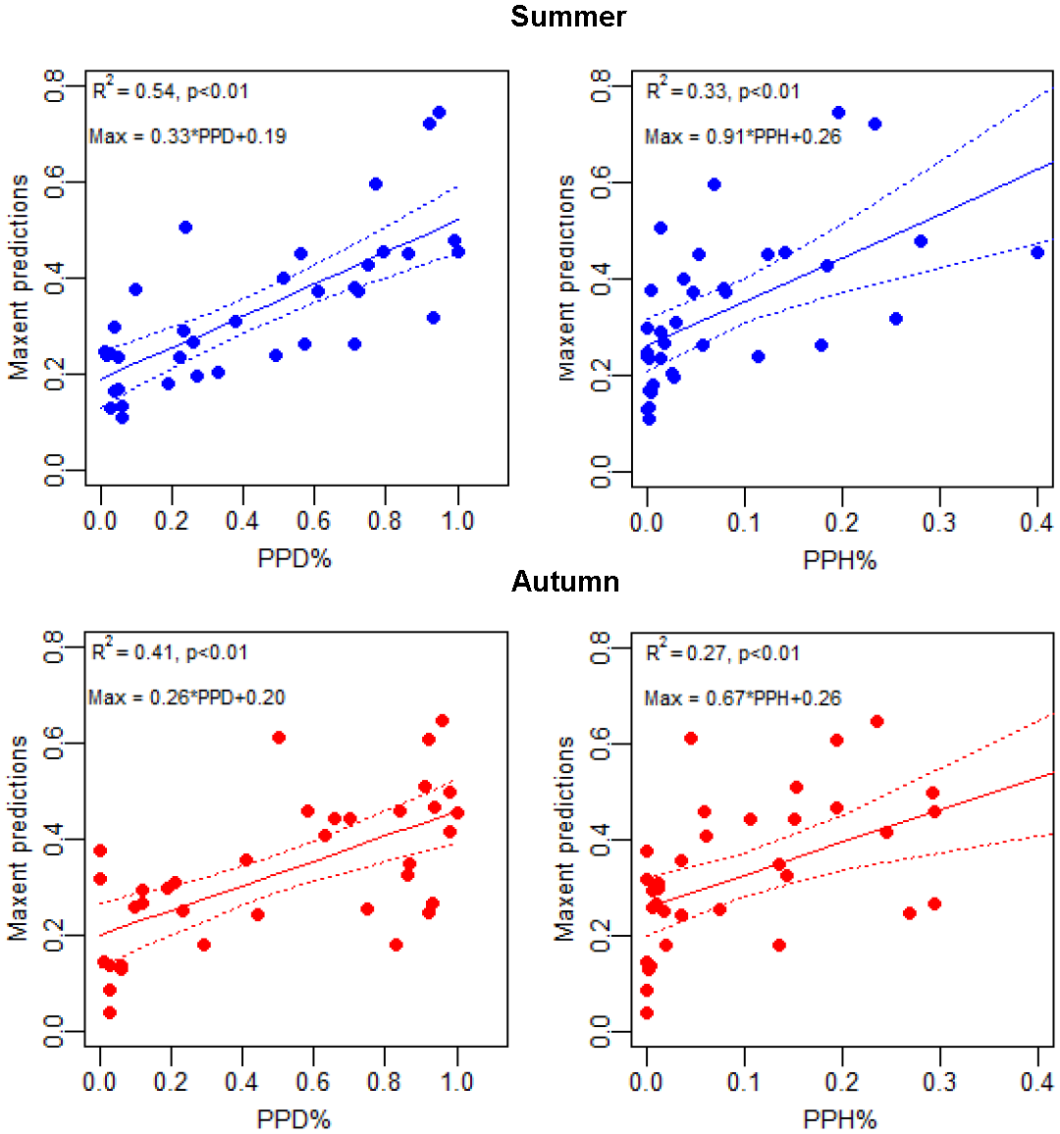
Correlation between data sets. MaxEnt predictions (mean of pixels included in a 1 km radius around each C-POD position plotted against % PPD (porpoise positive days) (left panel) and % PPH (Porpoise Positive Hours) (right panel) for the 36 C-POD positions by season. Pearson’s product-moment correlation curves show the relationship between the two datasets and R2 designates the variability described by the fitted line.

The relationship between the average MaxEnt predicted values within a 1km radius of the C-POD positions and the % PPD/PPH was described by Pearson’s product-moment correlation analysis (Fig 6). Both seasons display a significant positive correlation between MaxEnt predictions and the two C-POD data sets. The R2 values were relatively high for the PPD correlations (0.54 and 0.41, for summer and autumn respectively), and a little smaller for the PPH correlations (0.33 and 0.27 for summer and autumn respectively).

## Discussion

This study demonstrates that the two independent methods presented here correlate in the sense that high habitat suitability, based on satellite positions, provides high acoustic activity patterns in a marine mammal species. The large scale findings of the species habitat model MaxEnt were reflected in the small scale findings of the acoustic data set.

### Satellite telemetry

Enough satellite positions were available from harbour porpoises in the western Baltic Sea to model suitable habitats with MaxEnt for the two seasons summer (Jun-Aug) and autumn (Sep-Nov) with corresponding AUC values of 0.76 and 0.78, respectively. Even though AUC is not an absolute measure of model quality, it is an indication of the accuracy of the model and the value can be compared to other models. Elith [41] considered models with values above 0.75 as potentially useful and 0.80–0.90 as good.

Our MaxEnt model revealed putative suitable porpoise habitat hotspots in the western areas with a decreasing gradient to the east, reflecting the same pattern as the kernel densities during summer and autumn. The obtained AUC values for these two seasons were relatively high compared to those obtained in a similar study by Edrén et al. [11]. They used satellite positions from a larger area covering the Kattegat, Belt Seas and western Baltic partly overlapping with the present study area. The AUC values obtained by Edrén et al. [11] were 0.731 for summer and 0.697 for autumn, possibly reflecting the larger and more variable study area. Also, they did not find our study area to be very suitable during summer, but more so in autumn, yet with no clear gradient from west to east as observed here. These differences are most likely due to the smaller study area in the present study, capturing fine scale distribution patterns. Furthermore, the satellite data used by Edrén et al. [11] (1997–2007) contained fewer positions from our study area, as almost none of the animals tagged prior to 2008 moved that far east into the Baltic Sea. This may reflect a shift in the habitat use of porpoises. This was also shown by Benke et al. [52] where more porpoises were detected after 2008 in the eastern part of the German Baltic Sea.

The uncertainty of the model prediction is in general higher in the eastern part than in the western part of the study area, reflecting the fewer satellite positions here. All porpoises included in this study probably belong to the Belt Sea population as most of the animals were tagged here and their movements are within the population area [33]. The satellite data hence, does not tell us anything about the Baltic Proper population. The Belt Sea population has previously been shown to move south and east during summer and then move back west in the winter [6, 52]. This corresponds well with the results obtained here, reflecting the strong gradient from east to west.

The environmental variable that had the most explanatory power in our study was salinity in both seasons. The brackish water of the Baltic Sea arises from saline bottom waters from the North Sea mixing with fresh surface water originating from the Baltic Sea’s large catchment area, creating a salinity gradient decreasing from west to east and from surface to bottom [53]. Most of the satellite positions are located in the western part with decreasing numbers towards the east; this coincides with the gradient in salinity.

### Acoustic detections

Results from the acoustic study revealed a strong decreasing gradient in porpoise occurrence from south-west to the east. The results obtained here reveal more activity in the German part of the Baltic than what has recorded prior to 2008 [28, 54], similarly documented by Benke et al. [52]. Also, only very few animals have previously been spotted in the study area during visual observations during aerial and boat surveys [55–58]. Again, this indicates that the area has become more attractive for harbour porpoises within the past years in line with the increase in the number of positions from tagged animals.

### Model prediction vs. acoustic results

When inspecting the obtained results from the two methods, the gradient in the presence of harbour porpoises is clearly reflected both in the predicted model results, as well as in the acoustic porpoise detections, as also indicated by the significant linear correlations. Both show the same pattern of high occurrence in the south-western part of the study area decreasing towards the east, indicating that this area is mainly occupied by Belt Sea animals.

The best correlation between the two data sets was found between MaxEnt and PPD, both reflecting relative occurrences on a very broad scale. The obtained R2 values were relatively good when taking the different data sources into account, satellite observations from very few animals over a seven year period and the mean of environmental variables over a ten year period, with two years of acoustic detections collected with only partial overlap in time. A better correlation between the two data sets may have been obtained if the satellite data and the acoustic data were collected over the same years. For all correlations, there was an apparent offset in the MaxEnt predictions (starting at or above 0.2), which may reflect that all areas potentially have a value/suitability to porpoises (value above 0 in the MaxEnt model). However, as the density of porpoises in the study area is relatively low, the least suitable habitats are currently not used, as indicated by almost no porpoise detections on some stations. Alternative explanations for inconsistencies between the two data sets may be that Baltic Proper animals are present in the acoustic data, and if these animals prefer different habitats, or that there are too few satellite tagged porpoises included in this study to provide positions of all the preferred habitat types. Habitat models can only estimate the part of a species' niche that is actually captured by the ‘observation’ data. Their applicability to the unsampled areas is therefore dependent on the representativeness of the environmental conditions inherent in the input data [18].

MaxEnt model results are usually only trained on a subset of the “observation” data that enters the model for validation [15, 59–61]. In this study we were able to validate the model from a subset of the data, and also show that the model predictions were comparable to another independent data set from the same species, recorded in a completely different manner. This way of testing the model results may act as a more powerful validation [59]. Few other studies have verified MaxEnt model results based on independently sampled data for the target species [14–15]. Here we demonstrate that obtained MaxEnt model results were correlated with results from a large, independent and partly temporally overlapping data set on animal presence from the same area.

This study is the first to demonstrate similar patterns in these two independent methods; modelling of suitable habitats based on satellite tracking and passive acoustic recordings. In a similar way, Sveegaard et al. [62] used vessel based towed acoustic surveys to validate high density areas for harbour porpoises identified by satellite tracking. Hence, both satellite tracking and acoustic recordings seem reliable for monitoring relative distributions of a marine mammal species such as the harbour porpoise. In addition to providing reliable results on relative distribution, satellite tracking has a large spatial extend and may be used to identify individual habitat use, yet is very dependent on sample size. Passive acoustic monitoring has a limited geographical extend but provides long time series of variations in relative occurrences. Thus, the choice of method in future studies should depend on the specific goal, availability and time line of each project.

## Supporting Information

S1 Table C-POD recording periods. Successful data acquisition of the 36 C-POD stations included in the study. Data coverage is variable due to loss of equipment, malfunctioning equipment or loss of battery power. Also, the day of deployment/retrievement of equipment was excluded from the analysis, to only include full days.
(TIFF)
