# Supplementary figures and images for: Comparing Distribution of Harbour Porpoises (Phocoena phocoena) Derived from Satellite Telemetry and Passive Acoustic Monitoring
Source: PLoS One. 2016 Jul 27;11(7):e0158788. doi: 10.1371/journal.pone.0158788 (PMC4963116; doi:10.1371/journal.pone.0158788)

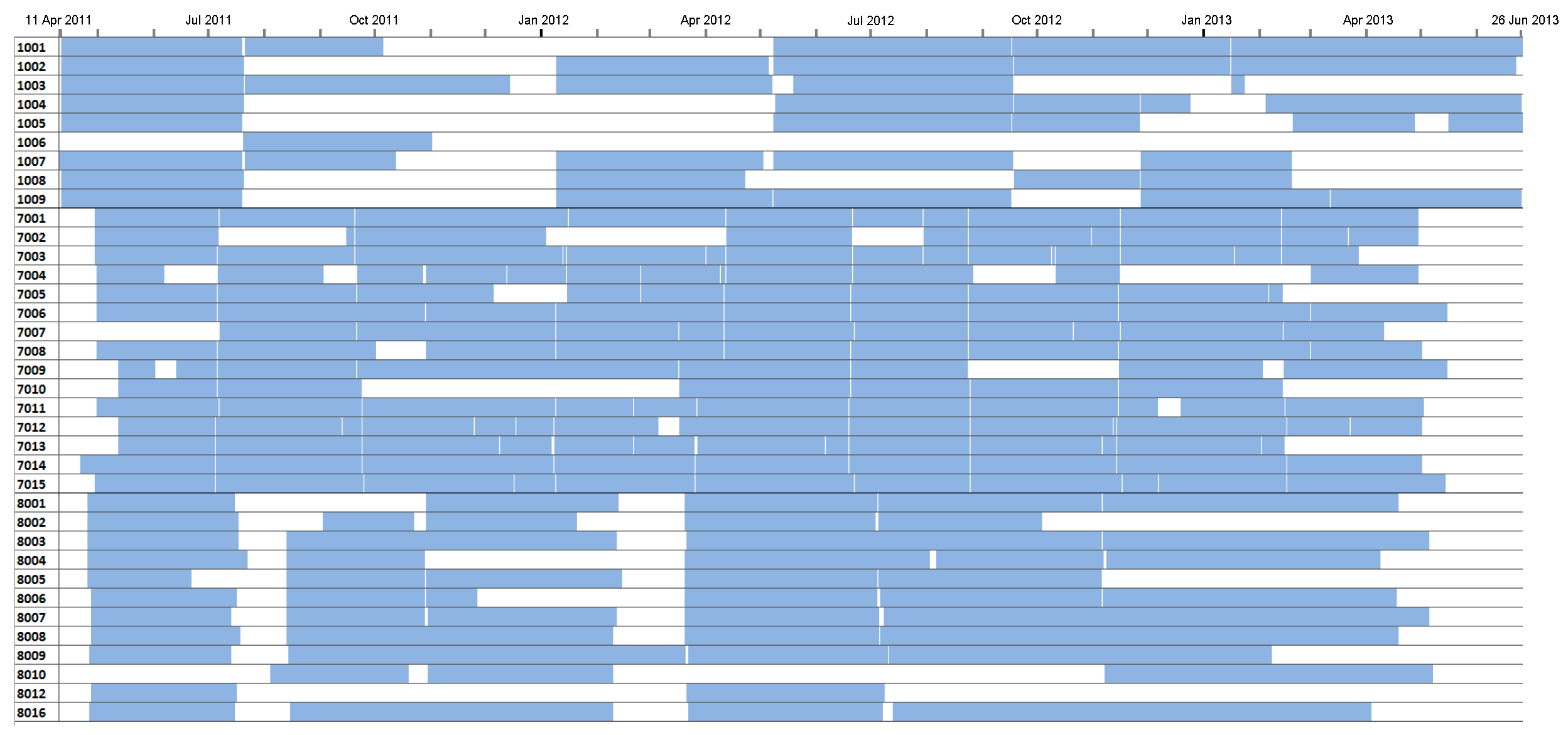

Supplement: S1 Table — Successful data acquisition of the 36 C-POD stations included in the study. Data coverage is variable due to loss of equipment, malfunctioning equipment or loss of battery power. Also, the day of deployment/retrievement of equipment was excluded from the analysis, to only include full days. (TIFF) [file pone.0158788.s001.tiff]
